# Epigenetic variation in light of population genetic practice

**DOI:** 10.1038/s41467-025-55989-6

**Published:** 2025-01-25

**Authors:** Sarah A. Mueller, Justin Merondun, Sonja Lečić, Jochen B. W. Wolf

**Affiliations:** 1 Division of Evolutionary Biology, Faculty of Biology, LMU Munich, Planegg-Martinsried, Germany; 2 Department of Microevolution and Biodiversity, Max Planck Institute for Biological Intelligence, Seewiesen, Germany; 3 Department of Ecosystem Management, Climate and Biodiversity, University of Natural Resources and Life Sciences, Vienna, Austria

**Keywords:** Evolutionary genetics, Epigenetics, Genetic variation, Evolutionary theory

## Abstract

The evolutionary impact of epigenetic variation depends on its transgenerational stability and source - whether genetically determined, environmentally induced, or due to spontaneous, genotype-independent mutations. Here, we evaluate current approaches for investigating an independent role of epigenetics in evolution, pinpointing methodological challenges. We further identify opportunities arising from integrating epigenetic data with population genetic analyses in natural populations. Efforts to advance data quality, study design, and statistical treatment are encouraged to consolidate our understanding of the source of heritable epigenetic variation, quantify its autonomous potential for evolution, and enrich population genetic analyses with an additional layer of information.

## Abstract (web summary)

Epigenetic variation can impact evolutionary processes to varying extents depending on its transgenerational stability and degree of environmental influence. This Perspective discusses the potential for epigenetic mechanisms to be integrated into population genetic practice.

## Evolution, epigenesis, and epigenetic inheritance

Evolutionary biology is concerned with transgenerational change. Prevailing evolutionary theory conceptualizes the underlying process as an interplay between five central parameters acting on genetic variation: mutations of the DNA backbone generate genetic variation, which is depleted by random genetic drift, reorganized by recombination and redistributed by migration (for definitions of italicized terms, see the glossary in Box 1). A subset of genetic variation translates into heritable phenotypic variation^1,2^ where it is filtered by selection. Over the last decades, progress has been made in understanding the underlying *epigenetic mechanisms* that orchestrate the construction of phenotypes from information encoded in the genotype^3^. Examples include non-coding RNAs (lncRNA, piRNA, siRNA, miRNA), structural protein templating (prions), and *chromatin modifications* (e.g. *DNA methylation*, histone modification)^4^. These self-perpetuating somatic inheritance systems can establish somatic molecular memory by controlling “the interaction of genes and their products […] which bring the phenotype into being”^5 p. 242^.

In evolutionary biology, the focus is often not on *epigenetics* in the classical Waddingtonian sense (*epigenesis*) concerned with predictable ontogenetic change from single-celled zygotes to fully fledged, multi-cellular composites (‘genotype-to-phenotype’)^5–8^. Instead, we are mainly interested in the trans-generational effects of epigenetic mechanisms, which may themselves serve as a heritable substrate in the germline beyond the information encoded in the DNA blueprint^9,10^. *Epigenetic inheritance* has received considerable attention, challenging whether the traditional evolutionary paradigm relying on genetic variation as the source of heritable variation appropriately captures all aspects of evolution (extended evolutionary theory^11–13^). Importantly, only the fraction of *epigenetic variation* arising independently of the genotype (source), showing genealogical stability (inheritance) and causing phenotypic variation to be filtered by selection (consequences), can make an autonomous contribution to (adaptive) evolution beyond variation encoded in the genotype (orange path, Box 2).

Evidence for the evolutionary relevance of epigenetic variation, particularly 5mC DNA methylation, has been reviewed elsewhere^14–16^. In brief, it seems rather widespread in plants and fungi^14,17,18^. Research, mostly conducted on genetic models in the lab, provides clear evidence for spontaneous, random *epimutations* in DNA methylation^19–23^, and to a lesser degree for environmentally-induced epimutations^24^. Most importantly, transgenerational inheritance has been reported for both^25,26^, but seems far from frequent^27,28^. While there are documented phenotypic consequences of autonomous epigenetic variation^29,30^, evidence is still limited^21^. In animals, with a stronger, albeit leaky soma-germline separation^31–33^ and *reprogramming*^34^, transgenerational inheritance of autonomous epigenetic variation is expected to be more restricted and harder to prove^35,36^, although evidence exists^37–42^.

In this Perspective, we explore the potential of integrating population genetic approaches to the study of epigenetic variation. We make a case for natural populations as a valuable source of information, provide a critical reappraisal of current practice, highlight shortcomings, and identify fruitful areas of future research. The target audiences are evolutionary geneticists interested in expanding their research to epigenetic variation, as well as researchers focusing on epigenetic mechanisms in the lab who are looking to tap into natural variation.

Box 1 Glossary The epigenetic literature has undergone semantic transitions and shift in focus sometimes resulting in inconsistent and fuzzy usage of central terminology^6^. We therefore provide a glossary to clarify our understanding and usage of key terms. In the main text, terms are italicized at first mention.

**Chromatin** refers to the nucleoprotein complex including DNA, RNA, associated proteins and their chemical modifications. Originally defined as a cellular component by microscopy, focus has changed to its capacity in gene regulatory processes rendering it the ‘physiological form of genetic information’^3^. See e.g.^127^.

**Chromatin modifications** are chemical alterations of *chromatin* components establishing the possibility of a ‘code beyond the DNA base sequence’. While there is some inconsistency in the literature, we here include both histone modifications and DNA methylation. See e.g.^4,14,127^

**DNA methylation** is a prominent example of a *chromatin modification*. It refers to the chemical modification of cytosine residues with a methyl group which can affect various regulatory functions in the cell. In eukaryotes it tends to occur in a CpG (animals) or CpCpG, CpHpH, CpNpG (plants) context. See e.g.^128,129^

**Epiallele** denotes the state of an *epigenetic variant* of a certain data type at a given locus (e.g. 5mC methylation of CpG cytosine residues or histone acetylation, say H2BK5ac).

**Epigenesis** is an ancient concept dating back to Aristoteles describing the development from an initially simple, homogeneous cell (zygote) to a complex organism as a gradual process. It is opposed to the idea of preformation assuming that the structural complexities of the embryo are already performed in the gametes. See e.g.^6,7^.

**Epigenetics** is a field of research concerned with the processes governing ontogenetic development. A main goal is to gain a detailed understanding of the regulatory molecular mechanisms involved in cellular differentiation and phenotypic variation more generally. See e.g.^6,7^. **Ecological and evolutionary epigenetics** are terms loosely used to characterize a field of research investigating the possible contribution of *epigenetic mechanisms*, to ecological and evolutionary patterns and processes including e.g. phenotypic variation, local adaptation, niche breadth or reaction norms. To date, a center of activity has been to find correlations between variation in *chromatin modifications* and geographic or environmental contrasts.

**Epigenetic inheritance** is a component of *epigenetics* concerned with the transmission of information that is not directly encoded by the DNA base sequence. Transmission can, in principle, occur at several levels of integration: between mitotically dividing cell lineages within an organism (soma-to-soma), between somatic cells and the germ line within an organism (soma-to-germline) or across genealogies (germline-to-germline). *Epigenetic inheritance* in the narrow sense is restricted to the unit of the cell and can be mediated by molecular *epigenetic mechanisms*. In a broader sense, *epigenetic inheritance* extends to higher-levels dimensions such as developmental and behavioral interactions between parents and offspring, niche construction, cultural transmission^12,13,130^) or the microbiome^131^. Here, we exclusively refer to the narrow-sense definition. *Epigenetic inheritance* across generations can be classified as intergenerational, if within the reach of paternal effects, or truly transgenerational if exceeding the F2/F3 generation of the paternal/maternal lineage, respectively. Transgenerational epigenetic inheritance is restricted to germline-to-germline, whereas intergenerational epigenetic inheritance can include components of soma-to-germline. See e.g.^2^.

**Epigenetic mechanisms** are molecular or other processes responsible for the transmission of information that is not encoded by the DNA base sequence. In a narrow sense these include cellular, non-DNA sequence-based self-perpetuating inheritance systems capable of establishing a molecular memory. Examples are chemical *chromatin modifications* (DNA methylation, histone modification), non-coding RNAs (lncRNA, piRNA, siRNA, miRNA), structural protein templating (prions) and their concerted interactions. In a broader sense, processes include parental behavior (brood care), niche construction or cultural transmission of information. See e.g.^3,9,14^.

**Epigenetic variation** is used in several different ways across the literature. Here, we define it broadly as the sum of differences in chromatin constitution between samples. This includes different scales of integration (within individuals, between individuals/populations/species), modes of transmission (soma-to-soma, soma-to-germline, germline-to-germline), types of chromatin modification (5mC methylation, histone modification) and different sources (genetic, environmental, spontaneous) (See Box 2).

**Epigenetic variants** refer to the all possible *chromatin modifications* of any type occurring in at least two different states, i.e. *epialleles*.

**Epimutation** refers to state alteration of any *epigenetic mechanism* introducing a novel *epiallele* in a given cell type at a given state in development. They are to be strictly separated from genetic mutations changing the state or order of the DNA base sequence. Epimutations can arise during somatic cell divisions or occur in the germline. Epimutations can arise spontaneously, be genetically or environmentally induced, and are the source of *epigenetic variation* (Box 2).

**Epiphenotype** is a term that emphasizes that epigenetic variation can be treated as a phenotypic trait. As any other phenotype, it needs to be well defined and amenable to standardized quantification (Box 3).

**Reprogramming** refers to tightly regulated erasure and subsequent re-establishment of *chromatin modifications* between generations. The extent to which *epigenetic variation* is reset by this process sets the boundary for the relevance of narrow-sense *epigenetic inheritance* for evolutionary change. See e.g.^34^.

Box 2 Sourcing epigenetic variation with evolutionary potential

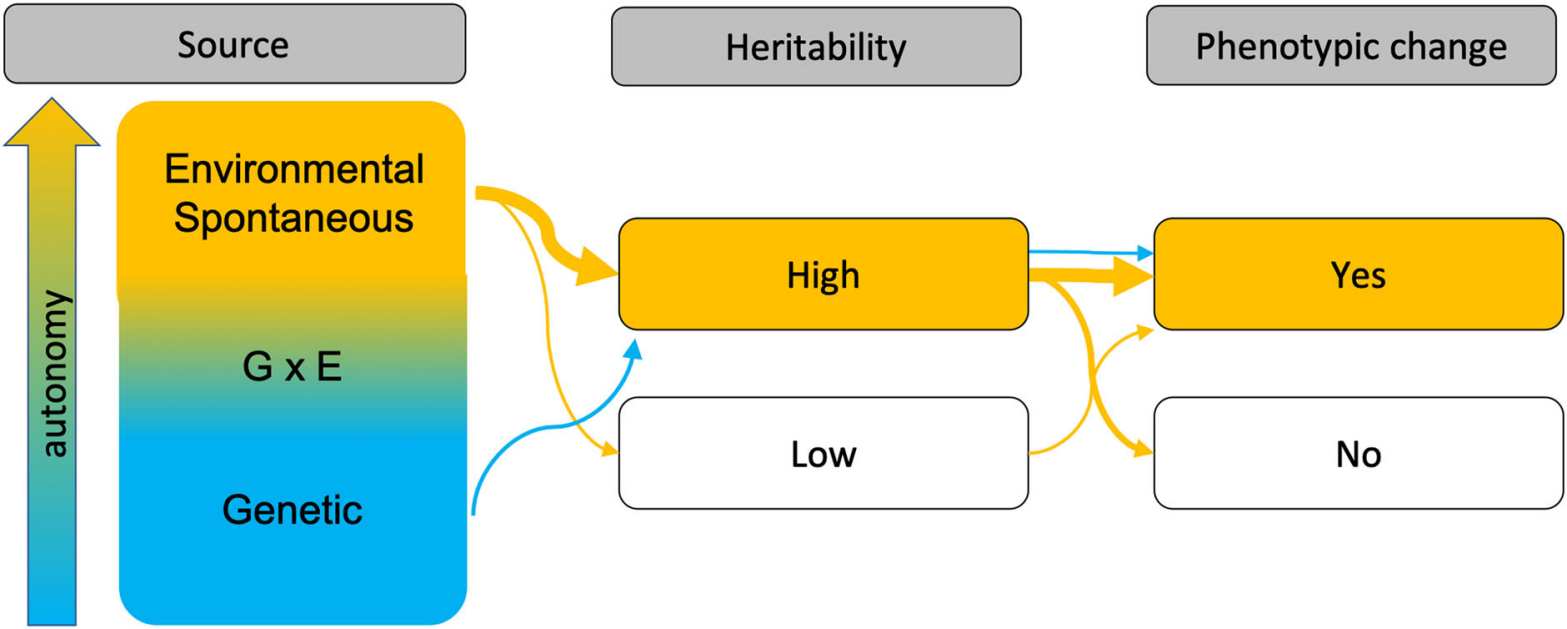

Terms relating to ‘epigenetics’ are often subject to ambiguity and have experienced semantic shifts^7,132^. *Epigenetic variation* is a particularly fuzzy term with inconsistent usage in the literature, see Box 1 for our definition. Epigenetic variation is thus an analogue to the term genetic variation which similarly encompasses different kinds of mutations (single-nucleotide mutations, structural mutations), which can be measured in different ways (allele frequency, heterozygosity, nucleotide diversity) and does not imply any evolutionary process per se (neutral, selected). A narrow, evolutionary definition of epigenetic variation would be best to only refer to the subset of inherited, non-genetically induced changes to chromatin modifications^8^. However, this poses a clear issue for researchers, as current measurements of epigenetic mechanisms are only an indirect proxy and would make it necessary to pinpoint the source before defining it as epigenetic variation^21,131,133^.

Epigenetic variation may be relevant to evolution through several pathways (see schematic). Epigenetic variants that are fully controlled by the genotype, implying a high heritability, constitute an interesting ‘pseudo-phenotype’ lying in between the DNA backbone and other phenotypes, which is worth studying in an evolutionary context (blue thin arrows). Non-heritable, spontaneous and environmentally induced epigenetic variants may provide the substrate for phenotypic plasticity that is of interest to a wide range of evolutionary questions (orange thin arrows). GxE interactions where genetic variants act in concert with non-heritable epigenetic variation can expand the phenotypic possibilities allowed by the genotype alone, modulate genetic phenotypic effects, and assume an intermediate position between full genetic control and environmental induction. All of these epigenetic mechanisms are important to evolutionary processes and should be explored. However, the main interest in epigenetic variation appears to be motivated by the possibility of an autonomous role of epigenetic variation to evolution^15,134^.

For the purpose of this Perspective, we, therefore, focus on the subset of epigenetic variation that may provide an autonomous, non-genetic substrate for transgenerational, evolutionary change (orange thick arrows). We are interested in chromatin modifications with high heritability operating independently of genetic variation, arising by spontaneous, random epimutations or induced environmentally (cf. Dobzhansky^135^). Epigenetic variation driven by gene-by-environment interactions assumes an intermediate position. A second filter is the stability of autonomous variation. While intergenerational epigenetic inheritance between cells is a necessary condition for cell differentiation during ontogeny, transgenerational inheritance beyond the F2 and F3 is required to leave an evolutionary mark independent of parental effects and genetic determination. Lastly, the relationship to phenotypic variation is a key aspect in determining the consequences of epigenetic variation for selection in natural populations, but not a necessary requirement for its evolutionary relevance (intermediate orange arrow).

## Epigenetic research in natural populations

Despite valuable baseline information from lab studies of model species, natural populations are the way forward to understand the role of epigenetic variation in the relevant ecological and evolutionary context^43^. To what extent do environmental stressors impact epigenetic variation? What proportion of environmentally or spontaneous *epialleles* are stably inherited in the wild? How does epigenetic variation mediate phenotypic plasticity or parental effects, and can this be maintained over generations when past stressors are no longer present? Do trans-generationally stable spontaneous epigenetic mutations evolve neutrally or can they spread in a population through selection? Is epigenetic variation with evolutionary impact associated with certain genes or found randomly along the genome? What is the role of epigenetic variation in speciation? Most of these questions can only be answered when the source of epigenetic variation is known (Box 2). While allowing for less experimental control, natural systems contain information to get at this question, as they allow us to partition variation across scales: between (related) individuals, populations, species and higher-order taxa, often across a multitude of environmental contrasts. This comparative approach is powerful. It has been successfully exploited to dissect the relationship between genetic and phenotypic variation^44–46^ and can be adapted for the context of trans-generational inheritance^47–50^.

## Current practice

We compiled information from 52 opportunistically sampled studies to identify current trends and challenges in the field addressing the above questions (Supplementary Data 1). We focused on studies investigating epigenetic variation in the form of 5mC methylation which still constitutes the most universally accessible and cost-effective chromatin modification^51,52^. We used several criteria to evaluate current standard practice regarding data quality measures, data analysis and statistical treatment, and integration into the theoretical framework. Overall, we feel that the field may have been running ahead too quickly, and should adjust aspects of data quality, analysis and experimental design differing greatly between plants and animals. Studies in plants generally have higher sample sizes, more standardized conditions and homogeneous sample types than animal studies; however, they lack taxonomic diversity in natural populations.

### Data quality

In regards to methodological decisions for DNA treatment, sequencing coverage, batch effects, and technical replicates, there appear to be no clear standards in the field, despite the direct impact on data quality (but see^53,54^ for advice). For instance, the importance of choosing homogeneous tissue unaffected by cell type composition is of central importance and has been largely overlooked. Only 44 % of the studies explicitly considered cell type homogeneity and the vast majority of animal studies used blood, known to respond to immune stress or other external factors^55^. Blood needs to be evaluated not only for the cell type composition, but also if it is reflective of methylation in other tissues^56,57^. Technical replication, which is vital to assess measurement error, evaluate performance of lab methods (enzymatic conversion vs. bisulfite treatment) and inform the upper bound of heritability, is rarely used (12% of studies), possibly due to the trade-off with adding more samples to increase statistical power. Finally, for all methods converting cytosine methylation signals to thymine excluding segregating genetic variation at CpG sites (C-T, A-G SNPs) is a simple, yet underused means to improve data quality (13% of applicable studies).

### Data analysis

We gathered information on the metrics used to quantify epigenetic variation. While studies use a variety of methods (MS-AFLP, MeDIP), the current standard is 5mC count data at single CpG sites (WGBS, RRBS, 61% of studies). Yet, there appears to be no clear consensus on the choice of data type nor the unit of choice, which will significantly impact downstream analysis (Box 3). A large number of studies analyze the proportion of methylated reads (40% of the studies) rather than raw read counts (12%), which contain more information^58^. Analyses based on single methylation loci make up 70% of the studies, while the rest integrated CpG-level methylation across regional levels to different extents. This choice is relevant from a statistical view point, since CpG locus count data can be approximated by a (beta)binomial distribution^58^, whereas integration across larger genomic areas might be better represented by other statistical distributions. We advocate that studies use both single loci and assess the intercorrelation of CpG loci within genomic regions (promoters, introns, exons, etc.) reducing the number of loci for downstream analysis^59^. Data-informed mechanistic models investigating the intercorrelation between CpG sites in a given genomic context will be important for guiding a biologically meaningful definition of *epiphenotype* (https://cran.r-project.org/web/packages/MethEvolSIM/)^60^.

A majority of examined studies (67%) attempted to find differentially methylated positions or regions (DMP or DMR, respectively) between two or more groups (i.e. treatments, populations, species, etc.). A subset of these were then related to gene expression (28% of studies) or were used for an *a posteriori* gene ontology narrative. However, caution is warranted in interpreting DMP/DMRs in natural populations. Unless explicitly addressed (Box 4), or at least in part controlling for genetic variation (23% of studies), we can only speculate on the source of observed differences. Moreover, samples sizes tend to be prohibitively low. Average sample size per treatment group was 18 (range: 2-336) which is substantially underpowered for detecting true positives, especially in differential methylation analysis (Box 5: simulations following ref. ^58^). This has important consequences for interpreting most standard analyses ranging from differential methylation to meQTLs which often invoke more complex models requiring even larger sample sizes to avoid overfitting. In the best case, conclusions are biased towards large-effect loci but may also refer to false positives.

In light of these limitations, we strongly recommend researchers conduct a pilot experiment to quantify the empirical distribution of effect sizes that can differ substantially between questions and systems. Power simulations can then provide insight into whether realistic samples sizes can be obtained to address the question at hand. While in most cases sample sizes in the hundreds or thousands will be required to detect small differences between variables of interest, a lack of power does not necessarily invalidate every study with moderate samples sizes. For example, clustering of large-effect loci in regions of elevated genetic divergence can still be informative on the underlying processes shaping epigenetic variation^57,61^. Moreover, where site-by-site analyses are strongly underpowered for small effect differences, other methods incorporating multivariate statistics^58^, machine learning^62^, or averaging across many genes in the genome^63^ can still extract useful information on the factors shaping epigenetic variation.

Box 3 How to measure epigenetic variation?

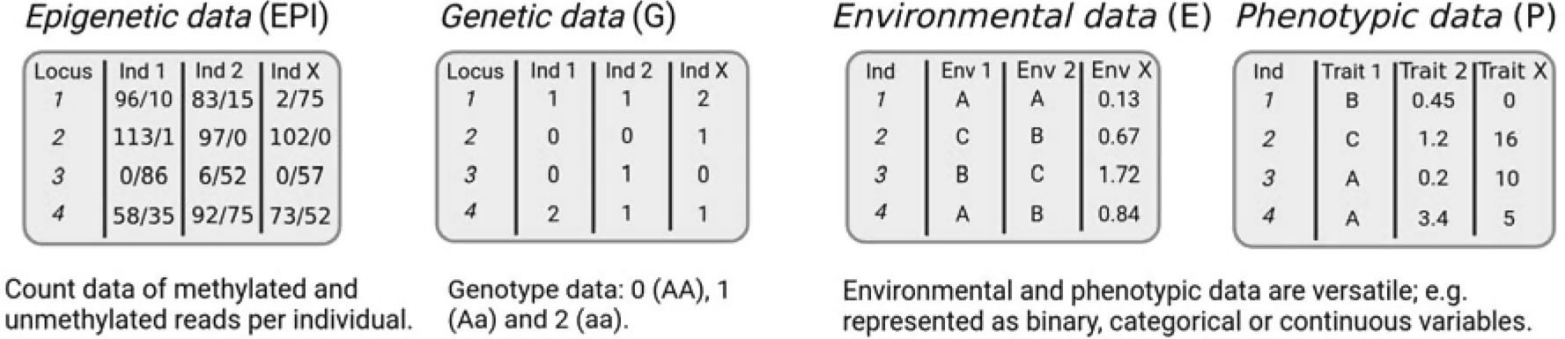

In contrast to the fully controlled conditions in the lab, research in natural populations embraces variation across levels: epigenetic, genetic, environmental and phenotypic. We therefore advocate for generating and combining data of all types for subsequent analyses in a joint statistical framework (see Box 4).

All data can be considered random variables that differ by type (categorical, continuous), measurement error and best-fit statistical distribution. While properties of genetic, environmental and phenotypic data have long received attention, the appropriate treatment of epigenetic data is substantially less clear. Epigenetic data can be viewed both as a phenotype (Box 4) or a genotype which will influence choice of units and measures for quantification of the raw data. The example below illustrates the associated challenges.
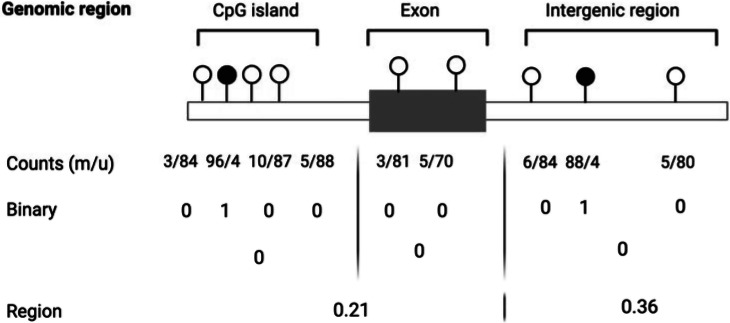

Here, we illustrate count data from RRBS or WGBS bulk sequencing of a tissue sample representing 5mC methylation as the number of methylated (m) and unmethylated (u) reads at the level of a single CpG site (single site). Such single-site data can then be treated as count data, proportions or categorical variables. Moreover, there are several ways to average CpG sites over functional categories such as CpG islands, exons, intergenic regions, or genomic windows of a fixed size (number of base pairs or CpG sites). The choice of the numerical scale, focal region, and averaging not only dictates appropriate downstream statistical treatment, but will impact biological conclusions. Until a better mechanistic understanding of chromatin modification can guide these decisions, sensitivity analysis may help to assess the contingency and robustness of inference.

Box 4 Epigenetic variation as a quantitative trait

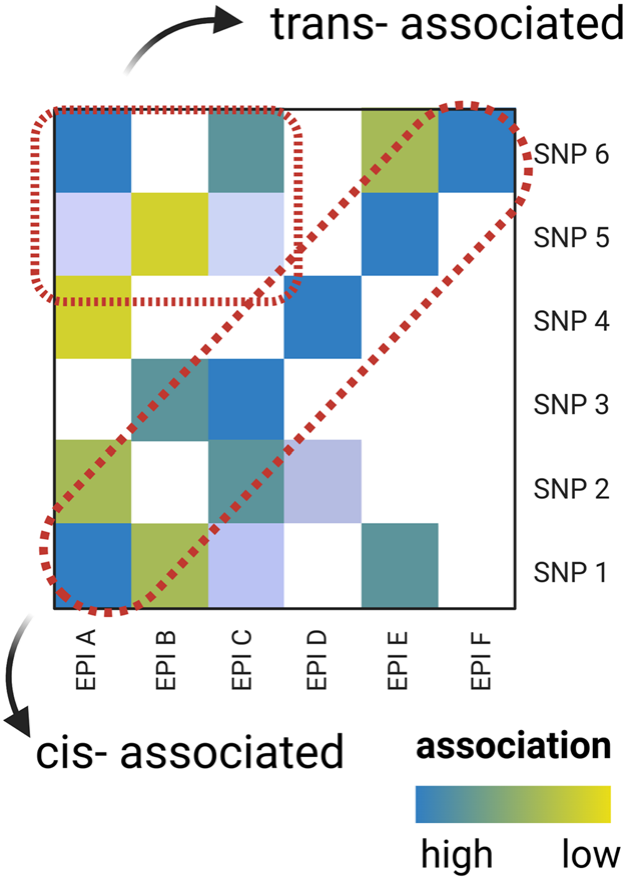

The study of epigenetic variation generally falls into standard quantitative genetic practice with all its promises, assumptions, and limitations (for summary see ref. ^136^ or consult refs. ^137,138^). We can define epigenetic variation as our phenotype of interest P.EPI. Following the animal model, the phenotype P of an individual *j* can be modeled as:

1 $${{{{\rm{P}}}}.{{{\rm{EPI}}}}}_{j} \sim {{{\rm{\mu }}}}+{{{{\rm{G}}}}}_{j}+{E}_{j}+{{{{\rm{\varepsilon }}}}}_{j}$$

where Gj is the genotypic effect of individual j relative to the population mean µ, Ej is an environmental contribution and ${{{{\rm{\varepsilon }}}}}_{j}$ is a residual term. Gj can further be divided into an additive genetic component Aj (breeding value) and dominance deviation Dj (Gj = Aj + Dj). The variance of the epi-phenotype V(P.EPI) can accordingly be decomposed into the environmental component V(E) and a genetic component (V(G) = V(A) + V(D)). Depending on the relatedness structure, heritability may only include additive genetic effects (narrow sense heritability V(A)/V(P.EPI)) or may also be informative about dominance variance (broad sense heritability, (V(A) + V(D))/V(P.EPI)). A note of caution, however, if assumptions are not met (e.g. additivity of genotypes, no genotype-environment covariation) the genotypic contribution will be underestimated (missing heritability problem^139^). Additionally, covariation between genetic and epigenetic variation need not imply a causal relationship of genetic variants affecting e.g. methylase activity. It may reflect linkage disequilibrium arising by other processes (see main text) adding an extra layer of complexity to tease apart the source of epigenetic variation.

An alternative to the above are regression models is an approach where the phenotype of interest depends explicitly on the genotype. The breeding value is then expressed by the sum over the individual effects of all *N* genome-wide markers (Eq. 2).

2 $${{{{{\rm{P}}}}.{{{\rm{EPI}}}}}_{j} \sim {{{\rm{\mu }}}}}+{\sum}_{k=1}^{N}{G}_{{kj}}+{E}_{j}+{{{{\rm{\varepsilon }}}}}_{j}$$

Here µ represents the intercept, G*kj* is the effect of the genotype at locus *k* in the focal individual *j*.

The schematic summarizes the effects of genotypes (SNP)_1-6_ on the phenotypic value of epigenetic loci_A-F_ (also reflecting the basic principle of meQTL analyses). Genetic loci in close physical proximity (cis-acting) to epigenetic loci are indicated by the same order of ascending index numbers (SNP1 is close to EPIA, SNP2 to EPIB, etc.).

Analyses utilizing Eqs. 1 & 2 can, in principle, inform us of the proportion of epigenetic variance that is either due to the environment or due to (heritable) genetic variation. Importantly, when treating epigenetic variation as a phenotype, we need to decide on how to quantify this phenotype (Box 3). Are we interested in a single CpG site, a CpG island or a multivariate representation of epigenetic variation across the genome? This choice will also determine the statistical treatment of this phenotype. The typical bimodal distributions of single CpGs can be categorized as a binary trait or a probability distribution that will require appropriate transformations. The correct choice is important and motivates further research, as deviations from the assumptions may introduce statistical bias.

Box 5 Sources of error in the analysis of epigenetic variation

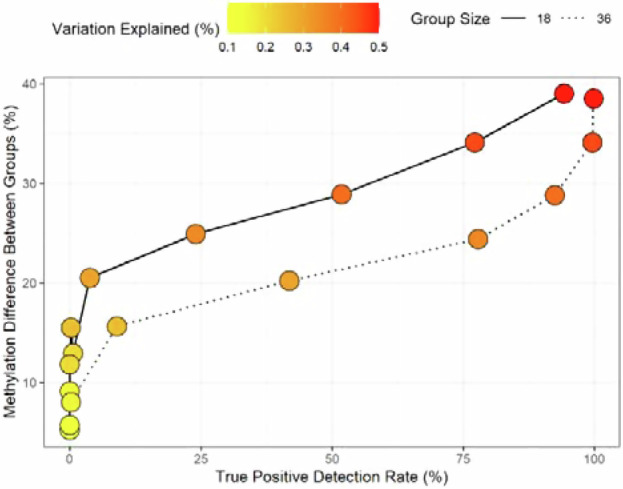

**True positive rate (power)**

Current studies using Eq. 2 (Box 4) or similar models identifying components of P.EPI tend to be underpowered and vulnerable to false positive inference. Consider a simple example of two populations of 18 individuals each where the populations differ by 10% methylation across all CpG loci. Assuming a beta-binomial distribution of 5mC count data and a 5% FDR detection rate and a simple binary explanatory variable (e.g. a single haploid SNP), essentially no true positive sites will be detected (see schematic). Even true positives differing up to 25% will only be detected approximately 25% of the time. Doubling the sample size (dotted line) increases power, but only markedly so for medium to large effect sizes (20% difference in methylation).

**False positive rate**

The false positive rate is of similar importance. While most studies correctly apply some form of multiple testing correction, genotyping errors are an underappreciated source for spurious associations. In the schematic below reflecting our own experience, one trans-acting SNP linked to an epigenetic locus is examined. Assuming high heritability, the expectation is that the homozygous states will show differing average methylation proportions. However, when one genotype is missing (in this case GG) and only one or two individuals are incorrectly genotyped as heterozygotes, this SNP locus will be associated to any locus in the genome where the incorrectly genotyped individuals have real or erroneous differences in methylation levels. In many cases, when mapped to a substantially larger number of individuals representing all genotypes, this association breaks down (rightmost panel). Such systematic false positive inference biases our view on the relative importance of single trans-genetic variation in shaping genome-wide epigenetic variation. Stricter minor allele frequency thresholds and outlier removal in methylation levels are the first measure. In the long run statistical models appropriately controlling for outliers are needed.

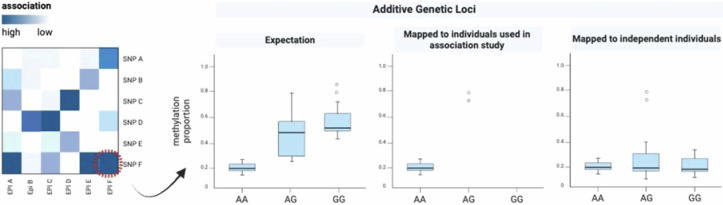

### Design

To assess the role of epigenetics in evolution, designs assaying transgenerational stability (at least generation F3) of epigenetic variation are crucial but remain extremely limited (only 11% of studies). This is understandable, since evaluating stability involves crossing designs which are not accessible for all study organisms. Leveraging divergent ancestry components (population stratification, hybrid zones) may constitute a viable workaround (see below). In any case, a study design should include reliable quantitative measurements from ecological, genetic and epigenetic data (Box 3). While examples embracing all data types exist^64–66^, only 43% of studies included independent genetic data, even less included individual-level WGS data (11%), which should no longer be an obstacle as individual-level genotypes can be called for single-nucleotide polymorphisms, and increasingly also for more demanding structural variants at rapidly decreasing costs^67,68^. The omission of genetic data compromises the ability to make inferences on the source and phenotypic impact of epigenetic mechanisms. Clearly, it is most beneficial to include DNA sequencing data from the very same individual and tissue to accommodate somatic mutations where possible.

## Decomposing the source of epigenetic variation

Population genetics is concerned with the processes shaping genetic variation across genealogical scales ranging from related individuals to populations and species. The quest for the source of epigenetic variation can benefit from drawing on established methodologies like quantitative genetics, variance decomposition in structured populations or genome scans. We can apply the same principles to (heritable) epigenetic variation and leverage population structure to investigate the processes shaping epigenetic variation. Nevertheless, challenges persist in refining experimental designs and downstream analyses. In the following, we summarize current methodologies and highlight opportunities for theoretical and empirical research to enhance the utility of epigenetic datasets.

### Quantitative genetic approaches

Regression-based approaches in quantitative genetics can play a role in decomposing the source of epigenetic variation (Box 4). One major challenge will be to find an appropriate definition for the epiphenotype that contains relevant biological information (Box 3) and reduces the number of pseudo-replication among all possible phenotypes (e.g. all CpG sites) in a genome.

**Fig. 1:**
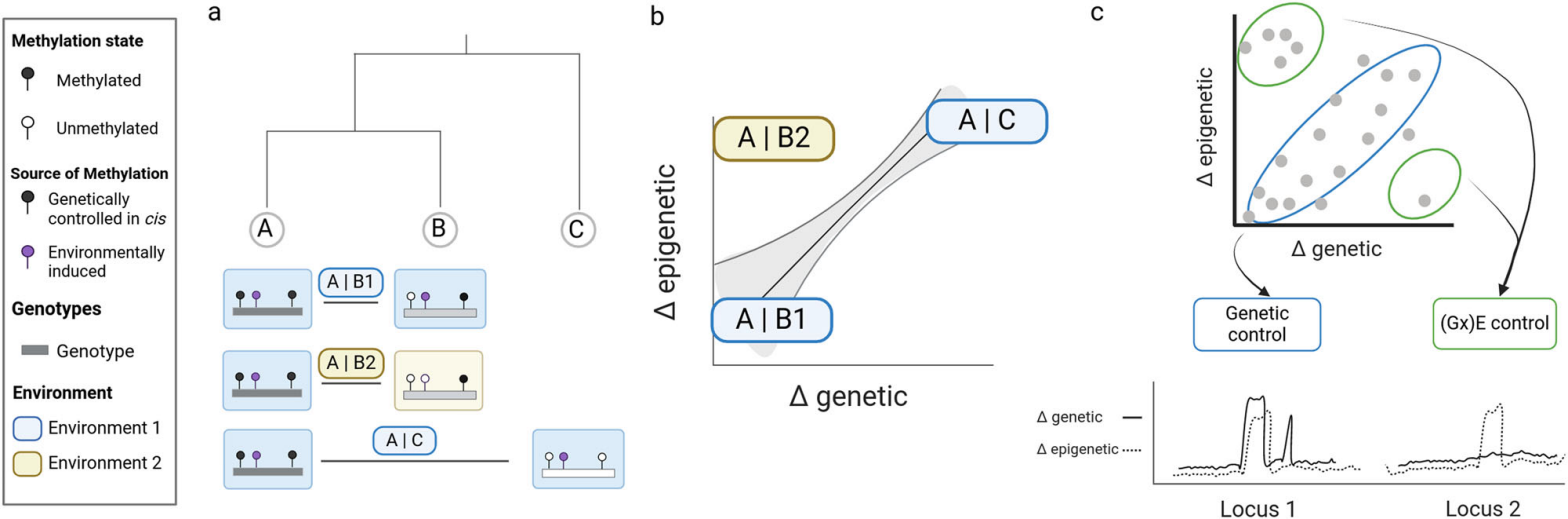
Making use of population divergence to analyze the source of epigenetic variation. Genealogical divergence (e.g. between individuals, families, populations, species) is typically reflected by the amount of difference in (heritable) genetic variation (Δ genetic). Contrasting heterogeneity in genetic and environmental divergence helps in assessing the degree of autonomous epigenetic variation (Box 2). **a** Three population samples with increasing genealogical distance are depicted (**A**, **B**), **C**). B1 (blue) and B2 (yellow) have the same genetic background, but are subject to different environmental conditions. **b** Associations between genome-wide genetic and epigenetic variation can be assessed using the correlation of distance metrics for genetic (Δ genetic) and epigenetic (Δ epigenetic) variation among genetic clusters, or through multivariate statistical approaches such as principle component analyses (PCA, not shown). For genetically controlled epigenetic variation, we expect to see a strong correlation between genome-wide metrics of genetic and epigenetic distance regardless of the environment. High correlation of genetic and epigenetic distance matrices between pairs of populations, and not the environment, may be a first indication of genetic control. **c)** While single-value comparisons per population provide a mean estimate across populations, window-based measures allow capturing variation along the genome. Capitalizing on the heterogeneity of genetic and epigenetic variation along the genome further allows to pinpoint regions of the genome under genetic and/or environmental control. The upper panel shows variation in genetic and epigenetic distance for the population comparison A | B2 at a regional scale along the genome. A point can refer to any genomic window in units of base pairs (or better units of centimorgans) here referred to as a locus. The lower panel shows an example of genetically controlled (left) and environmentally associated (right) epigenetic locus. Δ genetic is shown in blue, Δ epigenetic in red. Box 4 for corresponding statistical approaches.

In principle, the heritable component of epigenetic variation that is due to genetic variation can be estimated from epigenetic covariance of related individuals alone without the need for additional genetic data (Eq. 1). Independent genetic data, at base pair resolution, is recommended though, as models benefit from genomic estimates of realized relatedness, rather than pedigree-based estimates. Genetic data can also be used to estimate the additive effects of the genome directly (Eq. 2). This approach clearly faces the challenge of high dimensional data (more markers than individuals), compromising appropriate estimation of effect sizes. A number of primarily Bayesian approaches that assume prior architecture of the trait are in use^69^, and motivate exploration in the context of epiphenotypes. More commonly, genomic approaches are used to isolate genetic regions/loci that exert a strong effect on the state of the epiphenotype often by making use of crossing schemes (QTL analyses) or by exploiting naturally segregating variation in genome-wide association studies (GWAS). Such approaches are referred to by several names in the context of epigenetic variation (meQTL, MethQTL, epiQTL), but follow the same standard practice. In plants, this approach has been successfully applied to uncover the link between genetic and epigenetic variation at base pair resolution, mainly in *A. thaliana*^70–72^. They are also applied in human research^73–75^ and are gaining traction in natural populations of other species^36,76–78^. Methylation appears to be linked to genetic variation at varying rates depending on the study organism^47,61,64,79,80^. Despite successful application of quantitative genetic approaches, small sample sizes result in studies that are underpowered and susceptible to false positive inference due to erroneous SNPs and 5mC methylation calls (Box 5). Where small-scale studies fall short of rational use of limited resources, community attempts may be considered to generate a few large empirical datasets including both epigenetic and genetic data for the same individuals. Alternatively, integrative study designs and methods extracting relevant information from fewer individuals need to be developed alongside existing multivariate approaches (see below, cf. Fig. 1^58^).

Instead of using epigenetic variation as a focal phenotype, it can also be used as an explanatory variable to unravel variation in some other measurable trait. Formally, this would mean replacing P.EPI by any phenotype P.X and adding epigenetic variation as an additional explanatory component to Eqs. 1 and 2. Yet, it is challenging to isolate the independent contribution of epigenetic variation in such a situation, and few studies exist^81^. Importantly, the contribution hinges on the source of epigenetic variation and its stability. Transitory epigenetic variation induced by the environment could simply be subsumed in V(E). If epigenetic variation is fully heritable its effect should be captured by the genetic component V(G). Epigenetic variation will only make an independent, phenotypic contribution if it is environmentally induced or spontaneous and trans-generationally stable. This is an interesting topic and deserves further theoretical and empirical attention.

### Multivariate statistics

If we treat epigenetic variation as a multi-dimensional phenotype, then approaches like principal component analysis can integrate signals across the genome and extract useful information, which was carried out in 37% of studies we examined. Such multivariate methods provide a first look at patterns separating populations along the major axes of variation, like drift^82^ and/or selection dynamics^83^. This provides a useful starting point for global comparison with population stratification inferred from genetic data. It may even allow for locus-specific comparisons of epigenetic and genetic variation by identifying the loci of both data types with the highest loadings on each PC axis. Given that technical processing can have a large impact on epigenetic data (batch effects)^61^, we advocate the inclusion of technical replicates to identify and remove loci statistically related to non-target covariates or utilizing intra-experimental controls (e.g. including samples of variable developmental stages to isolate and eliminate age effects). (Partial) redundancy analyses (RDA) provide an additional option to quantify the contribution of genetic and environmental co-variates^84^, however, choice of co-variates can greatly influence the outcome^85^. Further investigation and best practice recommendations borrowing from other fields where high dimensional data is common are needed, particularly for the distributional properties of CpG data as well as the treatment of technically induced variation.

### Hierarchical population structure

Hierarchical multi-population comparisons constitute another way to decompose genetic, epigenetic, and environmental variance (Fig. 1a). Tests comparing sets of pairwise population matrices provide a means to investigate co-variance between two variables while controlling for a third. For instance, in evolutionary genetics research, the relationship of genetic and ecological distance is often of interest while controlling for the effect of geographic distance^86,87^. On the analytical side, partial Mantel tests have been a common choice, but multiple matrix regression methods promise more flexibility and appear to be better suited for this purpose^88^. In principle, such genome-wide queries can be adapted to genome-wide epigenetic distance between populations (Fig. 1b). Following the principle of genome-scans^89,90^ quantifying epigenetic variation along the genome alongside genetic data provides insight into the degree of local genomic covariation (Fig. 1c). Does high epigenetic differentiation coincide with regions of high genetic differentiation, or is it independent of background genetic signals? Such approaches could also be used to identify regions under environmental or cis-genetic influence. Yet, we are lacking appropriate distance metrics.

### Distance metrics

For genetic data, standard measures, such as F_ST_, d_XY_, d_A_, and multivariate statistics are readily available and are firmly rooted in population genetic theory with clear interpretation^82,90–93^. Yet, owing to a lack of suitable mutation and substitution models for chromatin modifications, we currently have no equivalent metrics for epigenetic data. F_ST_-related distance metrics such as Q_ST_ seem promising. Q_ST_ quantifies the additive genetic component of phenotypic variation and, in conjunction with F_ST,_ allows for inquiry into the selective or neutral forces underlying phenotypic divergence^94^. Lacking information on narrow sense trait heritability, P_ST_ is often used as an approximation with known limitations and pitfalls^95,96^. It seems appealing to apply the F_ST_-Q_ST_/P_ST_ framework to epiphenotypes as it places the variation we see in epigenetic data on the same scale as genetic data and allows to investigate whether epigenetic differentiation proceeds at the same rate as genetic variation (Fig. 1b). Elevated Q_ST_/P_ST_ suggests accelerated epigenetic divergence relative to genetic drift, which may hint at an environmental influence or selection acting on epigenetic variation. Several examples in natural populations already exist^65,79,80,97^, mainly using the proportion of methylation at a given loci as the input to calculate P_ST_.

Despite the promise, we need both empirical and theoretical research to accommodate specific features of epigenetic data. Importantly, this includes the definition of genomic scale for which the epiphenotype is considered (Box 3). Can we treat genome-wide epigenetic variation as a single phenotype to be compared to neutral, genome-wide differentiation reflecting genetic drift? Which genomic scale of cis-acting genetic variation is appropriate for comparisons with targeted epigenetic loci (e.g. specific CpG islands, promoter regions, etc.)? Can we develop models, analogous to genetic variation, to identify Q_ST_ outliers incorporating the effect of demographic change, rather than just picking loci from extreme percentiles? Specifically, which statistical distribution is best suited to estimate the variance components for calculating Q_ST_/P_ST_? How do we best incorporate covariance structure across epiphenotypes along the genome^98^, which will substantially change under cis- or trans-genetic control of methylation? Models and simulations predicting epigenetic change upon population differentiation should be particularly useful to get an intuition about the range of possible Q_ST_/P_ST_ under different conditions for the source of epigenetic variation (spontaneous, genetic control, environmental or GxE, Box 2). While teasing apart environmentally induced epigenetic variation may be possible, separating spontaneous epimutations from those under genetic control will prove more challenging, in particular under conditions favoring linkage disequilibrium (see below). We see a clear opportunity here to utilize theoretical and simulation approaches to examine the scenarios where this may – or may not – be possible.

## Integrating epigenetic variation in population genetic analyses

Characterizing the source of epigenetic variation is crucial to evaluate the autonomous potential of epigenetic variation to evolution. Yet, we also see an opportunity to broadly incorporate epigenetic datasets into population genetic approaches tracing evolutionary processes. Below, we explore potentially promising research directions.

### Linkage disequilibrium

It has long been proposed that phenotypic plasticity interacts with genetic evolution^99,100^. Adaptive plasticity enables individuals to enter novel environments with hitherto unexperienced selection pressures shaping (cryptic) genetic variation^101,102^. Depending on the conditions, populations in both original and new habitats may coexist, differentiate, or speciate^103,104^. Environmentally induced epigenetic variation could be one key mechanism mediating the interaction of adaptive plasticity and genetic variation^42,105^ – with consequences for how we interpret covariation between genetic and epigenetic variation.

Here, we illustrate the possible consequences of this interaction in the context of ecological speciation, where natural selection causes reproductive isolation between environmentally differentiated populations connected by migration. In brief, divergent selection reduces effective migration of alleles from barrier loci under divergent selection between populations. Linkage extends these genomic effects beyond the barrier locus, with the extent depending on selection strength and recombination rate^106^. As a consequence of this process, we observe statistical associations (linkage disequilibrium) between selected loci and neutral genetic variation both within and between populations, to various extents^90,107,108^. In principle, this process also applies to epigenetic variation. Assuming antagonistic pleiotropy^109^, where the alternate epiallelic state is respectively favored in different habitats, adaptive epimutations induced in the parental habitat reduce the fitness of migrants with an immediate effect on gene flow. If the epimutation is transitory, then the selective effects disappear in the next generation, resulting in no linkage with genetic variation. However, if epimutations exhibit some degree of transgenerational inheritance, they can come into linkage with surrounding (neutral) genetic variation or become coupled with other genetic or epigenetic variation subject to divergent selection^108^.

This influences our interpretation of the association between genetic and epigenetic variation. Thus far, we have treated epigenetic variation as a potentially heritable phenotype encoded by genetic variation. Associations between epigenetic state and genotype then suggest causal cis- or trans-genetic effects (Box 4). However, as illustrated in the previous paragraph, co-variation with genetic loci can also arise through selection and other mechanisms in the form of linkage disequilibrium^110^. Both theoretical and empirical research are needed to differentiate the processes inducing linkage disequilibrium between genetic and epigenetic variation and causal, additive genetic variation underlying epigenetic variation. Promising theoretical approaches exploring this interaction in an explicit population genetic framework are emerging, and are vital to inform empirical experimental design^111,112^. A first ad hoc approach to understand the source of linkage disequilibrium could consist of quantifying genetic-epigenetic associations within and among populations along a gene flow gradient under varying selection regimes in both natural and experimental settings.

### Hybrid zones

**Fig. 2:**
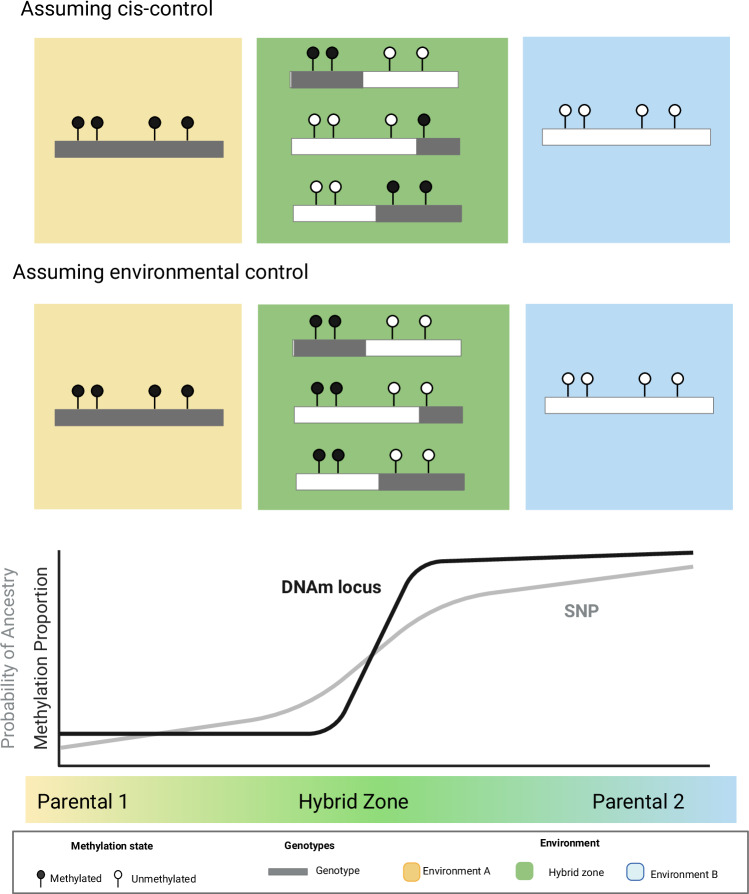
Epigenetic variation in hybrid zones. Hybrid zones allow to concomitantly study epigenetic variation and genetic ancestry and may help pinpointing the source of epigenetic variation (upper two panels). Cline analysis is a powerful tool to infer selection on both genetic and epigenetic variation and compare the two (lower panel). Geographic cline analysis characterizes the change of variation in genetic and epigenetic loci (here DNA methylation) on a spatial axis, whereas genomic cline analyses depict epigenetic divergence as a function of genome-wide ancestry.

Hybrid zones constitute a promising way of leveraging population divergence not only to decompose environmental and genetic components of epigenetic variation, but also to address the role of autonomous epigenetic variation in speciation. The use of hybrid zones has a longstanding tradition in evolutionary genetic studies^113^ and has increased our understanding of the processes governing population divergence^114^. By maximizing phenotypic and genetic variation and generating novel combinations thereof, they are suited to map the genetic basis of phenotypic variation while partially controlling for environmental variation in a geographically confined center of the zone^115^. For studies of epigenetic variation, hybrid zones confer similar advantages (Fig. 2). In the central part of the hybrid zone, environmental variation is often reduced relative to the parental core habitat, while genetic variation is maximized in the admixed genomes of hybrids, which can be represented as mosaics of ancestry blocks^116,117^. The genetic variance between ancestry blocks exceeds variation found within populations of a single species. As populations diverge, the number of fixed differences mixing in the hybrids increases our ability to pinpoint the effects of local, cis-genetic variation or genome-wide ancestry (trans-genetic variation). Increased allelic variance in hybrids can be particularly well exploited when considering allele-specific methylation, as this allows quantification of dominance deviation under cis-control, as well as identification of transgressive methylation levels indicative of trans-effects (genome-wide hybrid ancestry). Capitalizing on this idea, cis- and trans-genetic control of epigenetic variation has been quantified across the genome of flycatcher hybrids^63^.

Another promising avenue for exploiting information from hybrids may be to adapt geographic and genomic cline theory to epigenetic datasets and explore introgression dynamics of epigenetic and genetic loci^116^. Let us consider a geographic cline resulting from secondary contact of two populations that have previously diverged in isolation. In the center of the hybrid zone, we assume that the environment is rather homogeneous while genomes are admixed. Genetic variants sharing ancestry within a non-recombined segment of the genome will be characterized by elevated linkage disequilibrium. Epigenetic variants that are subject to cis-control should as a consequence also be in LD with one another and with the surrounding genetic variation. The same holds true for the fraction of spontaneous epigenetic variation that has accumulated differences during population isolation comparable to the surrounding genetic variation. Epigenetic variation under environmental or trans-genetic control should, however, not show this association. The former should show little to no variation, while the latter should reflect genome-wide ancestry (or ancestry at distant genetic loci). Expanding the rationale from the center of the zone to include a full transect of individuals from pure parental populations provides additional information. Genetic variants under divergent selection resist gene flow and are expected to show a steep cline^118^. Steep clines of single epigenetic loci may similarly reflect selection for trans-generationally stable epigenetic variation or simply reflect an underlying steep environmental gradient. The latter is less likely, while the former must be first separated from epigenetic variation maintained solely through genetic variants. The question of whether selection acted on the epigenetic locus or a genetic locus in LD is, however, difficult to answer, but may benefit from tissue-specific investigation.

This thought experiment illustrates two points: first, hybrid zones may hold key insight into the source of epigenetic variation and selection. Second, things are complicated, as processes can interact in intricate ways. Here, we can outline only the basic idea; theoretical research is needed to fill in detailed predictions. Evolutionary null models retracing the process of population divergence followed by secondary contact varying recombination, and genetic and epigenetic mutation rates could shed first light on the conditions necessary to distinguish the sources of epigenetic variation. Adding selection and coupling across regions of the genome may then help to understand the role of epigenetic variation in speciation. For simplicity, we here considered a geographic cline, but the rationale similarly extends to genomic clines, which hold promise for inferring the selection of epigenetic variation while controlling for genome-wide ancestry.

There are also a number of challenges on the empirical side. It remains to be explored how to best represent the change in epigenetic variation. Treatment as an epigenotype (binary or trinary) allows the calculation of epigenetic ancestry likelihood, analogous to genetic ancestry derived from genotype frequencies along the cline. An estimate of linkage disequilibrium would similarly require treatment of the epigenetic variation as a discrete genotype. Although cline analyses and measures of linkage disequilibrium can be conducted on a single epigenetic locus, analyzes will benefit from phased haplotype data embedding epigenetic information directly into (ancestry-informative) genetic background. With the advent of long-read sequencing data including information on chromatin modification such data can now be obtained. Despite substantial challenges, we see unexplored potential in ‘hybrid zone epigenetics’ and encourage both theoretical and empirical studies to adapt existing statistical approaches^116^.

### Demographic inference

Incorporating epigenetic variation into coalescent methods may be valuable for tracing recent demographic changes (i.e. bottlenecks, range expansion or contraction, or speciation) induced by changing climate or human-inflicted perturbations. CpG sites with clock-like behavior that are due to spontaneous epimutations may carry additional information on genealogies and may help estimate the timing of lineage divergence or reconstruct population demographic histories. Note, that such a ‘genealogical’ clock’ involves trans-generationally stable epimutations and is not to be confounded with an ‘ontogenetic’ clock tracing individual age^119^. A theoretical framework to predict the frequency distribution (site frequency spectrum) of highly mutable epimutations (mSFS) was developed by Charlesworth and Jain^120^ paving the way for statistical inference of neutral and selective factors influencing the methylome of *A. thaliana*^121–123^ and maize^124^. Going one step further, Sellinger et al.^125^ incorporated DNA methylation data into full-genome demographic inference. The conceptual novelty here is to infer the coalescent genealogy of a sample by integrating several markers in a genomic region: SNPs, but also additional markers with a higher mutation rate (e.g. 5mC methylation). This method extends the pairwise Sequentially Markovian Coalescent (SMC) models and allows us to infer: (1) the mutation rates of methylation/demethylation if the SNP mutation rate is known, (2) the relative importance of site- and region-level epimutation processes, and (3) the past demographic history of the population. The accuracy and resolution to reconstruct bottlenecks that occurred in the recent past is improved over estimates from genetic data (SNPs) alone^125^. Approaches leveraging spontaneous epimutations could be applied to resolve important evolutionary events as they can be reliably tracked even within one organism such as long-living trees or clonal organisms^126^. This relies on identifying neutral methylation sites unaffected by regional methylation and environmental changes. These examples are based on plant research where epigenetic inheritance is well established and are intended to motivate research in other taxa.

## Conclusions

The degree to which epigenetic variation makes an independent contribution to evolution is not yet clear. We advocate for research in natural populations complementing controlled experimental research in the lab. However, in order for epigenetic studies in the wild to make a lasting contribution to this question, they need to become more comprehensive by incorporating genetic, environmental, and phenotypic data in a rigorous statistical framework. Otherwise, the influence of the genetic sequence is likely to be underestimated, paving the way for epigenetic storytelling. The practical challenges are numerous and range from technical considerations and experimental decisions to finding meaningful metrics for epigenetic variation and appropriate statistical frameworks for data integration. Despite these challenges, we see the dual potential of this approach. The inclusion of epigenetic variation in population genetic methodology not only helps to assess to which degree epigenetic variation may or may not constitute an autonomous, non-genetic substrate for transgenerational evolutionary change. It also provides an additional axis of information for other questions in population genetics. The strongest approaches are those where we will be able to integrate genetic, epigenetic and environmental datasets with phenotypic measurements to understand the independent and joint impact of the DNA sequence and epigenetic modifications. In order to do this, we need to foster exchange between bench biologists and the empirical and theoretical population geneticists studying the evolutionary processes underlying (epi)genetic variation in natural populations.

## Supplementary information

Description of Additional Supplementary Files

Supplementary Data 1
