# Supplementary material for: Epigenetic variation in light of population genetic practice
Source: Nat Commun. 2025 Jan 25;16:1028. doi: 10.1038/s41467-025-55989-6 (PMC11762325; doi:10.1038/s41467-025-55989-6)
Supplement: Supplementary file 1 — Description of Additional Supplementary Files [file 41467_2025_55989_MOESM1_ESM.pdf]

Description of Additional Supplementary Files

Supplementary Data 1

Description: Literature review table
