# Supplementary material for: Epigenetic variation in light of population genetic practice
Source: Nat Commun. 2025 Jan 25;16:1028. doi: 10.1038/s41467-025-55989-6 (PMC11762325; doi:10.1038/s41467-025-55989-6)
Supplement: Supplementary file 2 — Supplementary Data 1 [file 41467_2025_55989_MOESM2_ESM.pdf]

Supplementary Table 1. A collection of opportunistically sampled articles on DNA methylation in (semi-)natural populations in both plants and animals. We summarize important categories relating to sampling, data processing, and data analysis: the species on which the study focuses, the kingdom, and the broad taxonomic level. If the study focuses on wild populations (wild), or if there is an experimental treatment done with wild populations (treatment), specific categories for lab organisms or cell lines is also given where applicable. The total sample size and the group size used for downstream analysis are reported. Sequencing method as reported in the study. If the study used standardized conditions (i.e., common garden), technical replicates, and if individuals were the same age at the time of sample collection, they were categorized into Yes/No. Cell type used and if this cell type is homogeneous or a correction for cell type was used is given. Next, if the study used independent genetic data, excluding SNPs called from bisulfite sequencing, and the type of genetic data included is reported. Inclusion of phenotypic, expression, and environmental datasets is given as Yes/No or a generalized category of the type of data used to compare across studies. If the studies correct for C-T SNPs in the methylation dataset, a Yes is given; for some methods this is not an applicable step. For animal studies, we report if the study mentions merging symmetric CpG sites, although not applicable to plants. For all, we report the minimum coverage threshold. For downstream analysis we categorized the CpG methylation level (loci or region). For region, we report the number of base pairs for window-based approaches or if the authors used the gene annotation to categorize CpG loci into functional categories. Data-type used reports the format of methylation for analysis (percent, proportion, counts, etc.). If the study was intra-generational we report to what generation; for more than F3 we report this as "Yes". The genealogical level reports at which level analysis was performed (families, groups, populations, species) as this can inform the expected methylation differences expected. Next, we report the types of analysis used, splitting these into three categories: IBE (clustering or distance-based analyses, like PCA, RDA, Mantel), differntial methylation analysis of loci of regions (DMC/DMR respectively), and genome-scan approaches (GWAS/meQTL/EWAS). We report where authors stated the statistical model used, the software, and statistical distribution to better understand current common methods in the field. Generally, not applicable (NA) and undeclared (UD).

Authors	species	kingdom	taxon broad	wild or lab population	sample size	group size for analysis	sequencing method	standardized conditions	technical replicates	age matched	cell type	cell type homogeneous	independent genetic data	phenotype data	expression data	environmental data	C-T SNP removal	merged CpG loci	minimum coverage filter	Loci based	Region based	data-type used	transgenerational	genealogical level	IBE (PCA,RDA, Mantel)	DMC/DMR	GWAS/meQTL/EWAS	statistical model	software used	statistical distribution
Riyahi et al. 2017 (A)	*Passer domesticus*	animal	bird	wild	84	10	MS-AFLP	No	Yes	No	pectoralis muscle	NA	No	morpological	No	climate data	NA	NA	NA	Yes	No	percent	No	subspecies	Yes	No	No	linear regression, AMOVA	SPSS	UD
Johnson and Kelly 2020 (A)	*Crassostrea virginica*	animal	mollusc	wild	80	10	RRBS	No	No	No	gill tissue	NA	RADseq	morphlogical	No	climate data	No	UD	10x	No	100bp	percent	No	populations	Yes	Yes	No	GLM	methylKit	binomial
Fargeot et al. 2020 (A)	*Gobio occitaniae and Phoxinus phoxinus*	animal	fish	wild	624	24	MS-AFLP	No	No	No	fin clips	No	Msats, poolseq	No	No	climate data	NA	NA	NA	Yes	No	UD	No	populations	Yes	No	No	distance based	Mantels tests, AMOVA,PCA	NA
Lea et al. 2016 (A)	*Papio cynocephalus*	animal	mammal	wild	69	3-39	RRBS	No	No	No	blood	blood smears	No	No	No	resource base	No	UD	UD	Yes	gene annotation	counts	No	environmental groups	No	Yes	No	binomial mixed effect model	MACAU	beta-bnomial
Mäkinen et al. 2021 (A)	*Parus major*	animal	bird	wild, treatment	28	10	RRBS	Yes	No	Yes	blood	No	No	physiological	No	lead exposure	No	UD	10x	Yes	No	counts	No	broods	No	Yes	No	GLM	methylkit/dss	binomial & beta-binomial
McNew et al. 2021 (A)	*Mimus parvulus and Taeniopygia guttata*	animal	bird	wild, treatment	21	10	epiGBS	No	No	Yes	blood	No	No	No	No	parasite exposure	No	UD	10x	Yes	No	counts	No	treatments	Yes	Yes	No	GLM	methylkit	binomial
McNew et al. 2017 (A)	*Geospiza fortis and Geospiza fuliginosa*	animal	bird	wild	42	6-15	MeDIP	No	No	No	blood, sperm	No	CNVseq	morphological	No	urban/rural	No	UD	UD	No	100bp	counts	No	species	No	Yes	No	GLM	edgeR	negative binomial
Sepers et al. 2021 (A)	*Parus major*	animal	bird	wild, treatment	26	~12	RRBS	Yes	No	Yes	red blood cells	Yes	No	No	No	brood size	No	UD	10x	Yes	No	percent	No	treatments	No	Yes	No	GLM	methylkit	binomial
Weyrich et al. 2016 (A)	*Cavia porcellus*	animal	mammal	wild, treatment	32	15-17	RRBS	Yes	No	Yes	liver, testes	Yes	No	body mass	Yes	diet	No	UD	UD	Yes	No	proportion	F1	offspring of treatment groups	No	Yes	No	Fisher's exact test	R	NA
Nilson et al. 2021 (A)	*Oncorhynchus mykiss*	animal	fish	wild, treatment	68	9-15	MeDIP	No	No	No	sperm, red blood cells	Yes	CNVseq	No	No	rearing habitat	No	UD	<50 rea(	No	1000bp		No	environmental groups	No	Yes	No	GLM	edgeR	negative binomial
Gore et al. 2018 (A)	*Astyanax mexicanus*	animal	fish	wild	54	26	WGBS	Yes	No	Yes	eYes	No	No	eye dengeneration	Yes	No	No	UD	UD	Yes	gene annotation	UD	No	phenotypic groups	No	Yes	No	UD	QUMA	no assumed distribution
Heckwolf et al. 2020 (A)	*Gasterosteus aculeatus*	animal	fish	wild, treatment	106	~12	RRBS	Yes	No	Yes	gill tissue	NA	Yes	No	No	salinity	Yes	UD	10x	Yes	No	counts	F2	populations	No	Yes	No	GLM	methylkit	binomial
Alvarado et al. 2015	*Camponotus floridanus*	animal	insect	wild, treatment	160	~20	LUMA	Yes	Yes	Yes	whole body	No	Candidate loci	body size	Yes	genome-wide DNA methylation	NA	NA	NA	No	genomewid e value	percent	No	treatments	No	No	No	Pearson correlation	Prism	NA
Lindner et al. 2021	*Parus major*	animal	bird	selection lines	54	~18	RRBS	Yes	No	Yes	blood	No	No	laying date	No	No	No	UD	10x	Yes	No	counts	No	selection lines	No	Yes	No	GLMM	LME4QTL	binomial
Sepers et al. 2023 (A)	*Parus major*	animal	bird	wild, treatment	286	~61	epiGBS2	Yes	No	Yes	blood	No	No	No	No	cross-fostering	No	Yes	10x	Yes	No	percent	No	treatments	No	Yes	Yes	GLM	DSS; MatrixQTL	beta-binomial; UD
Le Luyer et al. 2014 (A)	*Oncorhynchus kisutch*	animal	fish	wild	40	~10	RRBS	No	No	No	white dorsal muscle	NA	No	No	No	rearing habitat	No	UD	10x	No	1,000bp	UD	No	treatments	Yes	Yes	No	dbRDA, GLM	methylkit	binomial
Wang et al. (A)	*Acomys cahirinus*	animal	mammal	wild	29	~14	WGBS	No	No	No	muscle	NA	WGS	No	No	No	No	UD	UD	No	UD	percent	No	populations	Yes	Yes	No	GLM	DSS	beta-binomial
Cossette et al. 2023 (A)	*Sorex cinereus*	animal	mammal	wild	48	2-16	HorvathMammal MethylChip40	No	No	No	liver, tail, foetal	No	No	morphological	No	No	NA	NA	NA	Yes	No	beta-values	No	populations	No	No	Yes	GLM	limma	normal distribution
VerNAz et al. 2022 (A)	*Astatotilapia calliptera*	animal	fish	wild	35	~12	RRBS, WGBS	Yes	No	Yes	liver	No	No	No	Yes	habitat	No	No	UD	Yes	50bp	UD	F1	populations	Yes	Yes	No	MANOVA, GLM	DSS	beta-binomial
Jaffe et al. 2016 (A)	*Homo sapiens*	animal	mammal	wild	526	190-336	IllumiNA Array	No	Yes	No	ESCs, NPCs, neurons	Yes	WGS	schizophrenia	Yes	No	Yes	NA	NA	Yes	gene annotation	proportion	No	phenotypic groups	No	Yes	Yes	GLM	minfi	UD
Hu et al. 2021 (A)	*Gasterosteus aculeatus*	animal	fish	wild	94	11-64	RRBS	Yes	No	unclear	fin	No	No	No	no	no	Yes	UD	10x	Yes	No	count	Yes	population, family	No	Yes	Yes	LMM, power analysis	methylkit, MatrixEQTL, pyLMM	binomial
Skinner et al. 2014 (A)	*Geospiza, Camarhynchus, Platyspiza*	animal	bird	wild	10	~2	MeDIP	No	No	No	blood	No	CNV assay	No	no	no	NA	NA	No	No	tiling array	presence/ absence	No	species	no	Yes	no	Z-test	BS-genome	NA
Carja et al. 2017 (A)	*Homo sapiens*	animal	mammal	wild	34	5	IllumiNA Array	Yes	No	No	lymphoblastoid cell lines	Yes	SNP array	No	Yes	No	NA	No	NA	Yes	No	percent	No	population	Yes	No	No	LM, permutation	NA	NA
Liu et al. 2022 (A)	*Anguilla anguilla*	animal	fish	wild	36	5	WGBS	No	No	No	Muscle, tail end	No	WGS	No	Yes	climate data	No	Yes	5x	Yes	DMR, TEs	UD	No	sampling location	Yes	Yes	No		methlykit	binomial, custom outlier stats
Siliman et al. 2023 (A)	*Ostrea lurida*	animal	mollusc	wild	18	9	MBD-BS	Yes	No	Yes	Muscle, tail end	No	2bRAD	No	No	No	No	No	5x	Yes	No	proportion	No	populations	Yes	Yes	Yes	LM	MatrixEQTL	UD
Meröndon et al. 2019 (A)	*Lynx canadensis*	animal	mammal	wild	95	24	epiGBS	No	No	No	Pelt	No	No	No	No	climate data	No	UD	5x	No	5,000bp	percent	No	populations	Yes	Yes	No	beta regression	R	beta
Gawra et al. 2023 (A)	*Crassostrea gigas*	animal	mollusc	wild and farmed	246	60	bisulphite exome capture	Yes	No	No	Flesh	No	No	heat shock	No	disease resistance	No	UD	8x	Yes	No	percent	No	populations, treatment based	Yes	Yes	Yes	linear regression	CpGassoc	UD
Boman et al. 2024 (A)	*Ficedula flycatchers*	animal	bird	wild	70	3	WGBS	No	Yes	No	Brain, heart, kidney, liver, testis	Yes	No	No	Yes	No	Yes	UD	6x	No	gene annotation	proportion	No	species and hybrids	Yes	Yes	No	BSmooth	BSmooth	binomial
Smith et al. 2016 (A)	*Etheostoma olmstedi + 16 darter species (genera Etheostoma, Ulocentra, nanostoma*	animal	fish	wild	213	3-15	MS-AFLP	No	No	Yes	ovary	No	Yes	Yes	No	climate data	NA	NA	NA	Yes	No	genotype	No	interpopulation , interspecific	Yes	No	No	LR	standard population genetic software	dominant genetic locus
Feiner et al. (2022) (A)	*Daphnia magna*	animal	arthropod	laboratory	84	3	WGBS	Yes	No	Yes	whole specimen	No	No	fitness proxy	No	No	No	UD	5x	Yes	No	proportion	Yes	family	No	Yes	No	LMM	methylkit	LM betabinomial overdispersion
Carvalho et al. (2023) (A)	*Timema cristinae*	animal	insects	wild	24	2	WGBS	No	No	size match(	whole specimen	No	WGS	No	Yes	host-plant use	Yes	NA	No	Yes	No	percent, count	No	interpopulation , ecotype	Yes	Yes	Yes	LMM	MACAU	binomial
Baldanzi et al. 2017 (A)	*Talorchestia capensis*	animal	arthrophod	wild	150	30	MSAP	No	No	Yes	whole body	No	No	No	No	No	NA	NA	NA	NA	NA	presence/ absence	No	populations	Yes	No	No	PERMANO VA	PRIMER 6	UD
Whitaker et al. 2018 (A)	*Acipenser fulvescens*	animal	fish	wild	25	12	MS-AFLP	No	Yes	No	Blood	No	Yes	migration, morphological	No	No	NA	NA	NA	NA	NA	presence/ absence	No	populations	No	No	No	PERMANO VA	VEGAN	UD
Wogan et al. 2019 (A)	*Anolis cristatellus*	animal	lizard	wild	79	10	RRBS	No	No	No	Liver	Yes	No	No	No	climate data	No	No	10x	Yes	gene annotation	UD	No	populations	Yes	No	Yes	PCA + distance	PCADAPT	uniform
Thorson et al. 2019 (A)	*Potamopyrgus antipodarum*	animal	snail	wild	30	NA	MeDIP	No	No	No	Foot (muscle)	Yes	No	morphological	No	habitat	No	No	No	undelc	No	coverage	No	populations	No	Yes	No	GLM	edgeR	negative binomial
Hazarika et al. 2022 (P)	*Arabidopsis thaliana*	plant	angiosperm	greenhouse	106	NA	WGBS	Yes	No	Yes	Young plant	No	NA	No	Yes	No	NA	NA	NA	No	UD	genotype	Yes	generations, mutant lines	No	No	No	No DMRs	METHimpute	custom
Hofmeister et al. 2020 (P)	*Populus trichocarpa*	plant	angiosperm	wild	8	~4	MethylC-Seq	NA	No	No	Branches	No	NA	No	Yes	No	NA	NA	3x	No	DMRs	counts	No	individuals, mutation accumulation	No	Yes	No	one-sided z-test	Methylpy	UD
Schmitz et al. 2011 (P)	*Arabidopsis thaliana*	plant	angiosperm	greenhouse	16	2	MethylC-Seq	Yes	No	Yes	Leaf	Yes	NA	No	Yes	No	NA	No	4x	Yes	gene annotation	UD	Yes	families, mutation accumulation	No	Yes	No	linear regression	R (lm)	UD

van der Graaf et al. 2015 (P)	*Arabidopsis thaliana*	plant	angiosperm	greenhouse	58	NA	MethylC-Seq	Yes	Yes	Yes	Leaf	Yes	NA	No	No	No	NA	No	3x	Yes	gene annotation	genotype	Yes	families, mutation accumulation	No	Yes	No	custom	custom complex	UD
Kou et al. 2011 (P)	*Oryza sativa L.*	plant	angiosperm	clonal greenhouse	45	15	MSAP, bisulfite-PCR	Yes	No	Yes	Leaf	Yes	No	nitrogen deficiency	No	No	No	NA	NA	NA	NA	presence/ absence	No	treatments	No	No	No	NA	NA	NA
Han et al. 2018 (P)	*Zea mays*	plant	angiosperm	laboratory	38	1-6	SeqCap probe	Yes	No	Yes	3rd seeding leaf; callus for 1 cell line	No	No	No	Yes	No	No	NA	2x	Yes	target regions	proportion	No	tissue cultures	Yes	Yes	No	NA	BSMAP, pcaMethods	NA
Whittle et al. 2009 (P)	*Arabidopsis thaliana*	plant	angiosperm	laboratory	1088	200 / 96	NA	Yes	No	Yes	NA	NA	No	morphological	No	No	No	No	No	No	No	NA	Yes	treatments	No	No	No	ANOVA	SigmaStat 3.5	NA
Cubas et al. 1999 (P)	*Linaria vulgaris*	plant	angiosperm	greenhouse	39	~5	RFLP enzymes	Yes	No	Yes	young leaves	Yes	No	flower symetry	Yes	No	No	NA	NA	No	No	NA	No	strains	No	No	No	NA	NA	NA
Manning et al. 2006 (P)	*Solanum lycopersicum*	plant	angiosperm	glasshouse	UD	NA	CNR BSseq	Yes	No	Yes	fruit and leaf tissues	UD	No	No	Yes	No	No	NA	NA	Yes	No	percent	No	strains	No	No	No	NA	NA	NA
Dubin et al. 2015 (P)	*Arabidopsis thaliana*	plant	angiosperm	laboratory	150	113,150	MethylC-seq	Yes	No	Yes	whole plants	No	Yes	No	Yes	geographic data	No	NA	UD	Yes	No	UD	No	accessions	No	Yes	Yes	LMM	methylKit, LIMIX	binomial
Kawakatsu et al. 2016 (P)	*Arabidopsis thaliana*	plant	angiosperm	1001 genome project	1107	NA	MethylC-seq	Yes	No	Yes	rosette leaves	Yes	Yes	No	Yes	No	No	NA	UD	Yes	No	UD	No	accessions	No	Yes	Yes	LMM, eQTLepi	methylpy; LIMIX	UD
Sasaki et al. 2019 (P)	*Arabidopsis thaliana*	plant	angiosperm	1001 genome project	774	NA	MethylC-seq	Yes	No	Yes	leaf	Yes	Yes	No	No	No	No	NA	UD	Yes	No	UD	No	TE groups	No	No	Yes	LMM	methylpy; LIMIX	chi-square for GWAS
Gugger et al. 2016 (P)	*Quercus lobata*	plant	angiosperm	wild	58	NA	RRBS	No	No	No	mature leaves	Yes	No	No	No	climate variables	No	NA	10x	Yes	No	percent	No	individual trees	No	No	No	LMM	PyLMM	UD
van Moorsel et al. 2019 (P)	*Galium mollugo, Lathyrus pratensis, Plantago lanceolata, Prunella vulgaris, Veronica chamaedrys,*	plant	angiosperm	Glasshouse	47-96	5-12	epiGBS	Yes	No	Yes	frozen leaf tissue	Yes	Yes	morphological	No	No	No	NA	5x	Yes	No	percent	No	selection lines	Yes	Yes	No	GLM	DSS R package	beta binomial
Hagmann et al. 2015 (P)	*Arabidopsis thaliana*	plant	angiosperm	wild, w/ treatment	260	NA	WGBS	Yes	No	Yes	rosette leaves	Yes	WGS	No	Yes	rearing habitat	No	NA	3x	Yes	gene annotation	counts	No	accessions	No	Yes	No	HMM, LMM	model from Ionita et al.	beta binomial
Sellinger et al. 2023 (P)	*Arabidopsis thaliana*	plant	angiosperm	wild	10	NA	WGBS	No	No	Yes	whole plants	Yes	No	No	No	No	No	NA	UD	Yes	gene annotation	UD	No	accessions	No	No	No	HMM	eSMC2 R package	NA
Schmitz et al. 2013 (P)	*Arabidopsis thaliana*	plant	angiosperm	wild	152	NA	MethylC-Seq	No	No	No	leaf	No	Yes	No	No	No	No	NA	5x	No	gene annotation	percent	No	populations	No	Yes	Yes	LMM	EMMAX	binomial
